# Tumor Interstitial Fluid Formation, Characterization, and Clinical Implications

**DOI:** 10.3389/fonc.2015.00115

**Published:** 2015-05-26

**Authors:** Marek Wagner, Helge Wiig

**Affiliations:** ^1^Department of Biomedicine, University of Bergen, Bergen, Norway

**Keywords:** extracellular matrix, extracellular space, biomarkers, proteomics, tumor microenvironment, tumor extracellular fluid, interstitial space

## Abstract

The interstitium, situated between the blood and lymph vessels and the cells, consists of a solid or matrix phase and a fluid phase representing the tissue microenvironment. In the present review, we focus on the interstitial fluid phase of solid tumors, the tumor interstitial fluid (TIF), i.e., the fluid bathing the tumor and stroma cells, also including immune cells. This is a component of the internal milieu of a solid tumor that has attracted regained attention. Access to this space may provide important insight into tumor development and therapy response. TIF is formed by transcapillary filtration, and since this fluid is not readily available we discuss available techniques for TIF isolation, results from subsequent characterization and implications of recent findings with respect to fluid filtration and uptake of macromolecular therapeutic agents. There appear to be local gradients in signaling substances from neoplastic tissue to plasma that may provide new understanding of tumor biology. The development of sensitive proteomic technologies has made TIF a valuable source for tumor specific proteins and biomarker candidates. Potential biomarkers will appear locally in high concentrations in tumors and may eventually be found diluted in the plasma. Access to TIF that reliably reflects the local tumor microenvironment enables identification of substances that can be used in early detection and monitoring of disease.

## Introduction

The interstitium, or interstitial space, is a general term applied for connective and supporting tissues in the body. This space is located outside the blood and lymph vessels and parenchymal cells, and consists of two major phases: the interstitial fluid (IF) and the structural molecules comprising the extracellular matrix (ECM). The tumor interstitial fluid (TIF) is not only a transport medium for nutrients and waste products between cells and capillary blood, but also contains an abundance of substances that are either produced locally or transported to the organ by the blood circulation.

Cells have traditionally not been included in this concept of the interstitium (1). Cells in the interstitium, however, are active in continuous bi-directional cell–matrix interactions that result in microenvironmental changes, secrete substances to the IF and have important roles in initiating immune responses (2), and are a central element of the tumor interstitium. All of these are good reasons for including cells in the term “interstitium” here, notably those that are not organ specific, e.g., fibroblasts or immune cells, but rather an integrated part of the ECM. Whereas in previous years, the focus has been on the tumor cell *per se*, during recent years, there has been an increasing interest in the tumor microenvironment shown to be of significant importance for tumor growth and metastasis. The microenvironment consists of the insoluble elements of the ECM, the interstitial space with its non-tumor cellular elements (frequently referred to as stroma), and the fluid phase containing dissolved substances. While tumor microenvironment studies have mostly been on the stroma and the cellular elements of the tumor, we will focus on the fluid phase that has received less attention (3–5).

Here, we will review in brief the structure of the tumor ECM as a part of a general description of the tumor interstitium before we turn to the formation of TIF and techniques for fluid isolation of most relevance for the secretome, i.e., substances secreted by the tumor to the TIF. Our aim is to summarize recent studies on TIF where the focus has been locally secreted substances that will appear in the tumor at high concentrations, eventually appearing in the blood and thus reflecting processes at the tissue level. In the last part of the review, we will outline potential biological and clinical implications of new knowledge regarding secreted proteins and tissue microenvironment in tumors with respect to local signaling and the possible translation into new biomarkers. Although of interest in itself, fluids that are biologically more proximal to the disease site and thereby called proximal fluids (e.g., TIF) are also important elements in a more integrated approach toward biomarkers, also involving, e.g., tumor tissue, serum, and cancer cell lines (6). In a more extensive recent review, we have summarized literature on the formation of IF and TIF (7) and in another we have focused on the tumor secretome (8). Since the role of TIF as a source for biomarkers is an emerging and active field we will here give an update particularly focusing on recent developments in the area.

## The Tumor Interstitium and Interstitial Space – The Tumor Microenvironment

**Figure 1:**
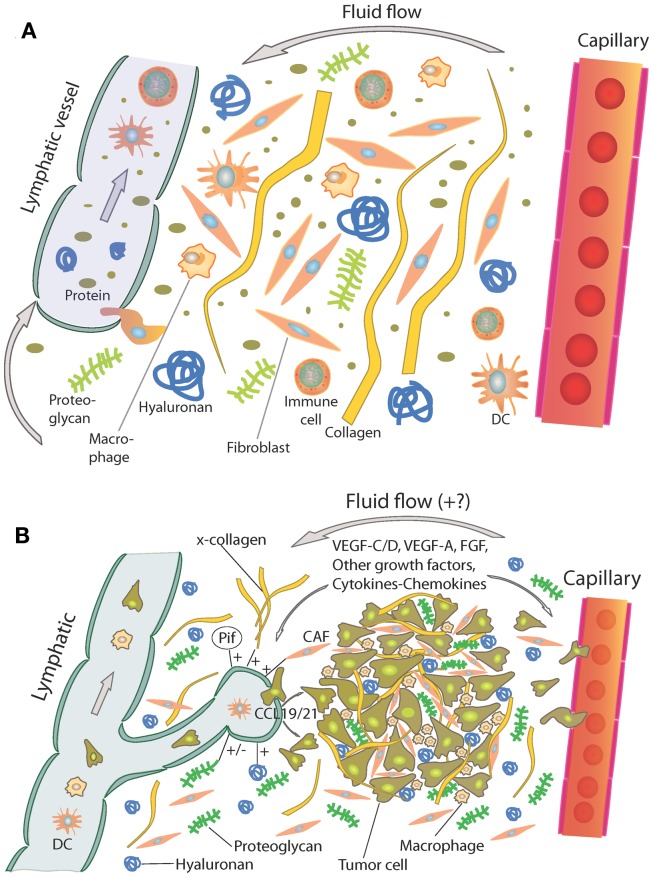
**(A)** Schematic overview of the interstitium with some of its major extracellular matrix components. Fluid containing plasma proteins and other solutes is filtered from the capillary percolates through the interstitium and is absorbed and thus returned to the circulation by lymph. In addition to proteins and solutes, immune cells migrate into lymphatic vessels and are transported to lymph nodes where they may initiate an immune response. Reproduced from Wiig et al. (128) with permission. **(B)** Role of the extracellular matrix and microenvironment in lymphangiogenesis in tumors. Growth factors and cytokines produced by tumor cells and stroma are transported by fluid flow and down a diffusion gradient to lymphatics and blood capillaries. Tumor and immune cells (expressing CCR7) are chemoattracted to and enter peritumoral initial lymphatics expressing CCL19/21. + (plus) and − (minus) denote stimulating and inhibiting lymphangiogenesis, respectively. x-collagen, crosslinked collagen; Pif, interstitial fluid pressure; CAF, cancer-associated fibroblast. Reproduced from Wiig et al. (128) with permission.

In general, the interstitium of normal tissue as well as tumors consist of a collagen fiber framework, a gel phase of glycosaminoglycans (GAGs), a salt solution, and plasma proteins. The structure and composition of the tumor interstitium/stroma have been covered in many recent comprehensive reviews, e.g., Ref. (9–15). A schematic picture of the tumor interstitium is shown in Figure 1. Because of the previous extensive literature on the topic, we therefore just discuss some salient features of importance for TIF pathophysiology here. As pointed out by Lu et al. (15), the ECM directly or indirectly regulates almost all cellular behavior and moreover the availability and activation of growth factors (14) and is therefore highly relevant also when discussing TIF.

Even though the tumor interstitium consists of the same components as the interstitium of normal tissues as depicted in Figure 1A, it has its special features that will be addressed briefly here. Compared with normal interstitium, the tumor stroma is “reactive” (9), involving *i.a*. an increased number of inflammatory cells, endothelial cells, and fibroblasts, which evolve with and provide support to tumor cells during the transition to malignancy (16). Macrophages are probably the most plastic among the inflammatory cells with tumor-associated macrophages (TAMs) serving as a paradigm for their functional polarization (17). In established solid tumors, TAMs contribute to angiogenesis, tumor invasion, and metastasis by producing proangiogenic factors such as vascular endothelial growth factor (VEGF)-A, epidermal growth factor (EGF), and IL-8, and proteases such as cathepsins, serine proteases, and matrix metalloproteinases (MMPs) (18). Therefore, an abundance of TAMs in the tumor interstitium is often associated with poor prognosis as revealed by analysis of pre-clinical and clinical data (18, 19). Progress has been made in defining signaling molecules underlying macrophage polarization *in vitro* (17, 20). Classically activated (M1) macrophages are induced by IFN-γ alone or in concert with microbial stimuli, such as lipopolysaccharide (LPS), or cytokines TNF-α and granulocyte-macrophage colony-stimulating factor (GM-CSF) and generally exert antitumoral functions (17). Conversely, IL-4 and IL-13 impose an alternative (M2) protumoral form of macrophage activation (17). Additionally, other molecules, such as macrophage colony-stimulating factor (M-CSF), can activate macrophages toward M2 direction (17). In solid tumors, bi-directional interaction between macrophages and the tumor interstitium shapes their phenotype. In response to various tumor- and stroma-derived cues, TAMs acquire M2-like state that shares a variable proportion of the signature features of M2 cells (17).

In contrast to macrophages, tumor-infiltrating cytotoxic T lymphocytes (TILs), including CD8^+^ T cells, are generally associated with good prognosis (21). CD4^+^ T cells, characterized by the production of IL-2 and IFN-γ, support CD8^+^ T cells and their high numbers also correlate with good prognosis (21).

Another myeloid cell population characterized by the immune suppressive activity has also been identified. These bone marrow-derived cells defined as myeloid-derived suppressor cells (MDSCs) are able to suppress CD8^+^ T cells activation through the expression of arginase (ARG1) and nitric oxide synthase 2 (NOS2), and induce the polarization of TAMs to M2-like state (22, 23).

Additionally, an increased number of fibroblasts that are called cancer-associated fibroblasts (CAFs) have a profound role with respect to tumor ECM composition and dynamics (13–15), resulting in a higher content of collagen, proteoglycans, and GAGs, notably hyaluronan and chrondroitin sulfate, e.g., Ref. (24–27). VEGF-A is a crucial inducer of reactive stroma formation (28) that may be secreted by inflammatory cells, by fibroblasts, or by the cancer cells themselves (29). The high levels of VEGF in tumors result in a high-microvascular permeability and extravasation of plasma proteins such as fibrin, again attracting fibroblasts, inflammatory cells, and endothelial cells (30, 31). These cellular responses resemble those of wound healing; although the process is dysregulated in the case of tumor stroma (32). It is established that stroma cells and fibroblasts are important for secretion of angiogenetic factors, e.g., Ref. (29), less is known on lymphangiogenic factors in this setting. Such secretion occurs, likely since inflammation has a pivotal role in tumor progression (33), and immune as well as tumor cells are important sources for lymphangiogenetic factors (34), again influencing the tumor stroma structure and function (Figure 1B). A very recent update on ECM biology is given in two particularly relevant reviews (35, 36).

## Tumor Interstitial Fluid Formation

As for normal tissues, the formation of IF in tumors is determined by properties of the capillary wall, hydrostatic pressures, and protein concentrations in the blood and interstitium according to basic principles for fluid exchange described by Starling more than a century ago (37). He suggested that the capillaries are semipermeable membranes, and that transcapillary fluid filtration is determined by the imbalance between oncotic (colloid osmotic) and hydrostatic forces. Later, important modifications have been introduced (38), resulting in the following expression for transmembrane flux applicable also to tumors, known as the Starling Equation:

(1) $$J_{\text{V}}=L_{\text{p}}A\left[\left(P_{\text{c}}-P_{\text{if}}\right)-\sigma \left(\text{CO}{\text{P}}_{\text{c}}-\text{CO}{\text{P}}_{\text{if}}\right)\right]$$

where *J*_v_ in the net capillary filtration, *L*_p_ is the hydraulic permeability of the capillaries, *A* is the surface area available for filtration, and σ is the capillary reflection coefficient. (*P*_c_ − *P*_if_) is the hydrostatic pressure difference between plasma in the capillaries (c) and IF, and (COP_c_ − COP_if_) represents the corresponding difference in colloid osmotic pressures. Solid tumors, however, have special features, notably a *P*_if_ that is elevated compared with normal tissues, as reviewed in, e.g., Ref. (39–41). Skin and muscle *P*_if_ are in the range of −2 to 0 (42), while pressures in tumors are positive both in experimental animals and humans, in the range of 10–40 mm Hg in the latter (39, 40). Interestingly, a dramatically high mean *P*_if_ of 99 mm Hg, and thus close to mean arterial pressure, has been observed in a model of pancreatic adenocarcinoma (43). The fact that tumor *P*_if_ is high may dramatically influence the delivery of therapeutic agents to tumors negatively, e.g., Ref. (41, 44) and has resulted in various efforts to counteract this effect and enhance drug uptake, as recently reviewed in, e.g., Ref. (45, 46).

Several factors may contribute to the high tumor *P*_if_, notably the tumor vasculature (39, 40), which due to the effect of VEGF and other factors is irregular, convoluted, and highly permeable (47) and have no pericyte coverage (48). Accordingly, there will be low restriction of protein and transcapillary water transport, resulting in high *L*_p_ and low σ in Eq. 1, and high interstitial “counter-pressure” to filtration synonymous to *P*_if_ (49). A low restriction to transcapillary fluid and protein transport and lack of functioning lymphatics in central tumor areas will result in a high COP_if_ (50, 51), the latter factor also contributing to the high tumor *P*_if_ (52, 53). Other factors contributing to the high tumor *P*_if_ would be intratumoral blood vessel compression due to solid stress due to growth (54), and direct effects of growth factors such as PDGF, TGF-β, and VEGF (40). Collectively, these special features of the tumor microcirculation contribute to a TIF deviating from the normal (7). Knowledge on these factors is of prime importance when attempting to overcome microenvironmental obstacles in therapy and to improve drug delivery to solid tumors (44, 55).

## Isolation of Tumor Interstitial Fluid

### Techniques for TIF isolation

When studying substances present in or secreted to the interstium, it is of prime importance to have methodologies that reflect the fluid microenvironment of the tissue cells, notably the local concentration of substances of interest to be able to decide whether substances are produced locally or brought to the respective interstitium by the circulation. In most tissues and conditions, IF is not readily available, and various methods have therefore been developed for IF isolation. Isolation of TIF represents a particular challenge due to the special properties of the tumor interstitium (see above), e.g., rich vascularization and high-cell content (4) and some of these challenges will be given special attention.

We have recently discussed more extensively available methods for IF and TIF isolation and evaluated their inherent strengths and weaknesses (7). Such an analysis is useful when deciding on a method for sampling of substrate for IF and, in particular, proteomic analysis. There have been no major developments in this field since our previous analysis (7, 8), and the reader is referred to these reviews for a more details. Available methods may be grouped according to whether the isolated fluid is native or derived, a fact that can be used to decide whether a substance is produced locally and a part of the secretome or comes from the general circulation. It is generally accepted that IF and lymph have the same composition and accordingly that IF and prenodal lymph both represent the fluid microenvironment for cells in a tissue (7). Tumor lymph collection might appear attractive, but even though lymph vessels are present in tumor tissue [for review see Ref. (52, 56–58)], these vessels appear to be non-functional, not draining any fluid (52, 53), and not cannulable, making lymph sampling inapplicable in tumors. Techniques that have been used in tumors are tissue centrifugation, tissue elution, ultrafiltration, and microdialysis (59), as depicted schematically in Figure 2 in Ref. (8).

### Tissue centrifugation

Tissue centrifugation (51) is one of the more recent methods developed to sample TIF for native fluid and secretome analysis. It was originally applied for cell-poor and collagen-rich tissues like cornea (60) and tail tendon (61), but it later turned out that TIF could be extracted by exposing tumors to an increased G-force. Methodological studies using the extracellular tracer ^51^Cr-EDTA have shown that provided application of a *g*-force of G ≤ 424 there is no dilution of extracellular fluid. Based on these and other validation experiments, we concluded that the isolated fluid was representative for TIF (51). The procedure has been used in other tumor models (62, 63), and was recently translated to human ovarian carcinomas (64) and validated using two “internal” markers, namely Na^+^ and creatinine, assumed to distribute predominantly in the extracellular fluid phase.

### Tissue elution

A much-used method for TIF isolation is tissue elution, originally introduced by Celis and co-workers as a method when searching for a substrate for biomarker analysis (65). With this technique, fresh biopsies isolated from women with invasive breast cancer are cut into small pieces (1–3 mm^3^), washed carefully, and incubated in phosphate buffered saline. The supernatant collected after 1 h elution is named tumor IF. Although TIF collected this way contained major serum proteins as might be expected, the general protein profile deviated strongly from that of serum.

A potential problem with the tissue elution method is that the peptides and proteins found in isolated fluid may derive from cell fluid released during sectioning for elution and thus be of intracellular origin. This may not be a problem when searching for biomarkers, but may make it very challenging to calculate the exact tissue concentration in order to decide whether a substance is produced locally or brought to the tissue by the circulation.

### Capillary ultrafiltration

Ultrafiltration, a technique mostly used for purification or separation of chemicals, has also been applied to sample tissue fluid after implantation of capillary probes *in vivo* (66). With this method, negative pressure is applied to the probe. The recovery for small molecules is ~ 100%, and the *in vitro* recovery for albumin 74–100% depending on sampling time (67). Membranes with MW cut-off of 400 kDa have been used to allow for collection of proteins in TIF. For tumors, the technique has also been applied for collection of TIF from fibrosarcomas in mice (68), and it has been claimed that the collected fluid directly reflects the tissue concentration (69). Even if a high MW cut-off membrane is used, the protein concentration in the ultrafiltrate is very low compared with that found with alternative approaches, calculated to be <1/2000 (7) of that in TIF of other tumors in mice (50). This is probably due to sieving of tissue proteins at the capillary membrane, in the tissue or at the tissue-membrane interface during ultrafiltration (7), and will be accentuated with increasing protein size. Ultrafiltration fluid will accordingly not represent TIF composition.

### Microdialysis

Microdialysis, originally developed for fluid sampling from the brain, is a method frequently applied for isolation of endogenous and exogenous substances from the extracellular space also in other organs. The technique has been used extensively to study TIF [for reviews see, e.g., Ref. (70–72)], although mostly in pharmacokinetic and pharmacodynamics studies (73–75). The underlying principle of the method is that of passive diffusion of substances across a semipermeable membrane. Although initially used for sampling of small molecules, microdialysis has during recent years also been applied to examine peptides and proteins in the extracellular fluid phase [for recent reviews see, e.g., Ref. (76–78)]. When applied for this purpose, the recovery of macromolecules in the dialyzate may, however, be very low (~1%) due to various physical restrictions (77). The dialyzate will thus not reflect the concentration and molecular size distribution of substances in TIF, a deviation that will increase with increasing molecular size. This fact notwithstanding, the technique has been applied in studies of peptides and proteins dissolved in TIF (59, 79), i.e., in a context where the movement of such substances to the dialyzate may be severely restricted. Microdialysis is likely more suitable for investigations of small molecules also in tumors, including the “metabolome” (80).

## Composition of Tumor Interstitial Fluid

### Characteristics of TIF

The composition of TIF has recently been addressed in a comprehensive review by one of us (7) and moreover in a recent review by Baronzio et al. (5) and is therefore summarized just briefly here. When compared with plasma and subcutaneous IF, TIF has a high P_CO2_ and lactate, and a low P_O2_ and pH (Table 1), with an ionic composition close to that of plasma (81). The interstitial acidity has been found to be related linearly to tumor size in rats, decreasing from pH of ~7.3–6.2 with increasing tumor mass up to 50 g (82). High-capillary permeability and dysfunctional lymph vessels (53) have been suggested as explanation for the relatively high TIF protein concentration and thus a high TIF COP, being ~80% of that in plasma and significantly higher than the corresponding ratio of 50–60% in subcutis (50, 51, 64). It is likely that tumor specific proteins are found in TIF at high concentrations.

**Table 1:** **Composition of interstitial fluid in tumors**.

Tumor type	Host	P_O2_ (mm Hg)	P_CO2_ (mm Hg)	P_CO2_ (mm Hg)	P_CO2_ (mm Hg)	pH	pH	pH	Lactic acid (mg/l)		Reference
		TIF	TIF	SIF	Plasma	TIF	SIF	Plasma (arterial)	TIF	Plasma	
Carcinoma (Walker 256)	Rat		79 ± 6	50 ± 2	31 ± 1	7.044 ± 0.044	7.341 ± 0.30	7.313 ± 0.041	12 ± 3	5.1 ± 4	(81, 129)
Chinese hamster lung fibroblasts	Mouse		76.9 ± 7.9			6.85 ± 0.05			20 ± 1.2		(130)
Carcinoma (Walker 256)	Rat					6.98 ± 0.13	7.30 ± 0.11				(82)
Colon adenocarcinoma (LS174T)	Mouse	8.3 ± 1.6				7.04 ± 0.02					(131)
Cervical cancer	Human	<10									(132)
Various	Human	<10									(133)

*TIF, tumor interstitial fluid; SIF, subcutaneous interstitial fluid*.

*Empty cells in table: value not determined. Reproduced from Haslene-Hox et al. (8)*.

Although to our knowledge not investigated directly, TIF conceivably contains a class of substances called matrikines (3). These are the result of limited enzymatic cleavage of numerous extracellular proteins and GAGs that exert biological activities (83, 84). Interestingly, their biological activity is usually different from their parent full-length molecules (84), a property that may be exploited in anti-cancer therapy (85).

Tumor interstitial fluid likely harbors extracellular vesicles (EVs) [also called microparticles, e.g., Ref. (86)] that have been isolated from most bodily fluids (87, 88). EVs have received considerable attention during the last years, shown by the almost exponential increase in published papers addressing this issue. Such vesicles are one likely component of the multifaceted TIF and are therefore just briefly considered here, but a recent broad and extensive review of the biogenesis, secretion, and intercellular interactions can be found in Colombo et al. (88). EVs are a heterogeneous population of cell-derived vesicles enclosed by a lipid bilayer with a diameter of 30–2000 nm released from cells that appear to be involved not only in normal physiological processes like tissue repair, immune surveillance, and blood coagulation but also have a pathophysiological role, including that of tumor growth and progression, e.g., Ref. (87, 89). There are three main classes of EVs; exosomes, microvesicles, and apoptotic bodies (87), and their classification are based on cellular origin, size, biological function, or biogenesis. A considerable increase in EV generation is, however, found in various pathological conditions, including inflammation and autoimmune diseases, vascular conditions, and malignancies as discussed in several comprehensive reviews, e.g., Ref. (86, 89–95). EVs may contain mRNA and microRNA, signaling proteins cytokines, and pro-thrombotic factors, and represent a network for exchange of intercellular information and thus paracrine signaling. In tumors, EVs are shed from tumor as well as stroma cells to the surrounding microenvironment. Although not shown, it is highly likely that IF contains EVs that are enriched in TIF. Interestingly, EVs have been used to monitor tumor therapy in real time (96), and have emerged as possessing therapeutic opportunities (87). Although a normal phenomenon, EVs also reflect pathological processes and is a likely source for biomarkers. As stated earlier (8), there are apparently no studies on content or composition of EVs in TIF and this topic ought to be addressed in future work.

### Concentration gradients in IF

Analysis of IF in normal tissue as well as tumors may enable the assessment of the quantitative importance of local production of, e.g., signaling substances and thus a better knowledge on pathophysiological processes at the microenvironmental level. Local production in the respective interstitium will appear as a higher concentration in IF than in plasma (P), i.e., IF/P > 1, since any solute transported across the microvasculature from plasma will result in an IF/P < 1.0.

An insight into such pathophysiological processes was given in a study on patients with acute myeloid leukemia. Iversen and Wiig (97) isolated bone marrow IF and could identify substances with a potential mechanistic role in leukemia development. Whereas fluid isolated from bone marrow repressed hematopoietic cell growth, there was no response to plasma. The IF repression effect was, however, lost by successful induction treatment, suggesting that the hematopoiesis inhibiting factor(s) was/were not present in this situation, an assumption supported by the observation of maintained repression in cases where the treatment was unsuccessful. The IF/P ratio of adiponectin and TNF-α exceeded 1.0, thus showing local production. The cytokine concentrations fell in patients that went into remission, there was, however, no corresponding reduction in plasma levels.

**Figure 2:**
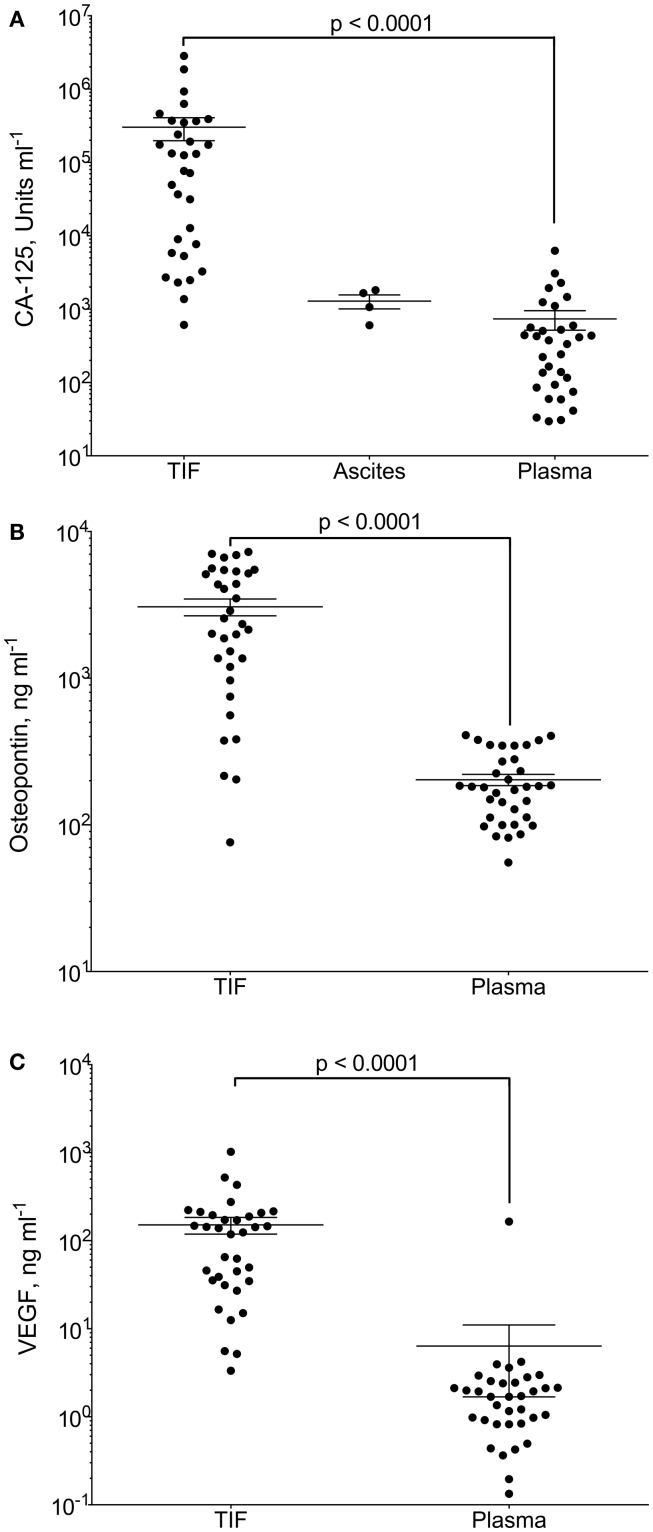
**CA-125, osteopontin and VEGF-A in tumor interstitial fluid (TIF), ascites, and plasma**. Concentration of **(A)** CA-125 (U ml^−1^); **(B)** osteopontin (ng ml^−1^); and **(C)** VEGF-A (ng ml^−1^) in TIF, ascites (for CA-125), and plasma from patients with epithelial ovarian carcinomas. Values are for individual tumors and also show mean ± SEM. ***p = 0.0001 (Wilcoxon matched pairs signed rank test). Reproduced from Haslene-Hox et al.(98) with permission.

Gradients between the tumor interstitium and plasma have been presumed for tumor specific proteins and are an assumption in most biomarker studies, as will be discussed in further detail below. In a recent study, we presented proof of this concept by isolation of native, undiluted, TIF by centrifugation from ovarian carcinomas (98). We assessed the TIF/P ratio for the known ovarian cancer biomarker cancer antigen (CA)-125 and the more general tumor markers osteopontin and VEGF-A. All three were significantly up-regulated in TIF relative to plasma (Figure 2). Not surprisingly, this finding was most pronounced for CA-125, having a TIF/P ratio ranging from 1.4 to 24,300, with a median concentration 194 times that of plasma. This study documents possible TIF/plasma gradients that may occur, and exemplifies the advantage of using TIF as a source for biomarker and therapeutic target discoveries.

As evident from the studies discussed above, there will be concentration gradients between TIF and plasma for substances secreted by tumor and stroma cells. Of interest, there may also be local gradients within the interstitium because of the flow of IF toward the lymphatics that may be of considerable physiological and pathophysiological importance. In experiments in mice supported by *in vitro* data, Swartz and collaborators have shown that tumor cells generate autologous gradients of CCR7 ligands by secreting them into the interstitium under the influence of slow IF flow toward lymphatics [reviewed in Ref. (99)], and have named this phenomenon autologous chemotaxis (100). Based on these data they also suggest that IF and lymphatic flow in the tumor microenvironment result in mechanical changes to the tumor stroma and affect the immunity of the tumor. These examples of studies on IF and lymph demonstrate the importance of focusing at the local microenvironment. An implication would be that tissue fluid should be used to interrogate local pathophysiological processes if available, and also that that the TIF and lymph subproteomes may deviate from plasma.

## Tissue-Specific Substances Originating from TIF

### TIF as a vehicle

In the remaining part of the paper we will focus on the application of TIF as a vehicle or substrate for substances and tumor-specific proteins that are secreted (i.e., secretome) to the extracellular and thereby the IF phase. In this way, we may gain knowledge on biological processes that may be translated into diagnostic, therapeutic, and prognostic use. This inevitably leads into the topic of biomarkers that will be discussed in the context of TIF. Biomarkers have become a vast and continuously expanding topic during the last years, and we will try to limit ourselves by having a special focus on ovarian carcinomas. This notwithstanding, our discussion may have relevance for other solid tumors and even leukemias as discussed above (97).

Although the main focus in this section is TIF as a source for biomarkers, alternatively, analysis of medium abundant proteins isolated from TIF but carried to the interstitium by filtration of plasma may also reveal properties of the ECM. Plasma proteins in the IF may also be considered as protein probes with a given size and charge that are distributed in the interstitium depending on properties of the ECM. With this rationale, we recently developed a novel approach, involving the exact determination of albumin concentration and mass in IF and tissue eluate by HPLC and thereafter, expressing the corresponding numbers relative to albumin for a set of probe proteins assessed by quantitative proteomics on unfractionated IF (101). We later used this method to determine plasma protein distribution volumes in human ovarian and endometrial cancer using normal postmenopausal ovarium as control, and found that the distribution of abundant plasma proteins in the interstitium depends markedly on hydration and ECM structure (102). Interestingly, these data can be used in modeling of drug uptake, and give indications on ECM components to be targeted to increase the uptake of macromolecular substances, and is an alternative translational use of the TIF-analysis data. Although not the main focus of the study, a number of earlier proposed biomarker candidates were detected in increased amounts in malignant tissue, e.g., stathmin and spindlin-1, again suggesting that IF, even when unfractionated, can be a valuable source for tissue-specific proteins (102).

### TIF and secretome as source for biomarkers

During the recent years, there have been rapid advances of mass spectrometry techniques enabling the identification and quantification of thousands of proteins in biological samples. This fact, together with a corresponding improvement of bioinformatics, enabled the search for biomarkers with high throughput. In spite of the considerable effort that has been invested in this search, identification of candidates fulfilling all the requirements of a biomarker has been sluggish, e.g., Ref. (103–106). Actually, as concluded in a recent review (106), the “inconvenient truth” is that no biomarker developed by proteomics has proven to be beneficial for cancer patients.

Clearly, blood or plasma is the preferable material for a diagnostic test. In spite of significant technological advances, the present proteomic technology, however, has limited power to detect a “needle” (low abundance disease biomarkers) in the “haystack” of high abundance plasma proteins. To reduce this problem, a possible strategy in a biomarker search might be to increase the relative abundance of disease-associated proteins by moving “upstream,” to samples more proximal to the primary disease site (103, 105–107).

As recently shown in our study on the established biomarker CA-125 (98) and most likely applying to all tumor-specific biomarkers (104, 108, 109), there will be high concentrations locally in the diseased tissue. The concentration will, however, be reduced in the perimeter of the lesion and the substance in question will be substantially diluted in blood. Accordingly, proximal fluids like TIF appear to be attractive substrates (107). Naturally secreted proximal fluids, as cerebrospinal fluid, saliva, urine, and nipple aspirate fluid, have been substrates in proteomic discovery studies [e.g., reviewed in Ref. (110)]. Examining TIF, however, will allow studies of shed and secreted proteins in tissues and conditions where natural secretion does not occur, e.g., in tumors. TIF is the best substrate to study proteins secreted by cancer cells and other cells confined in the tumor microenvironment, i.e., the cancer *secretome* (111, 112).

Cell line supernatants and proximal (i.e., close to the anticipated source) biological fluids have been the two main substrates for studies of the cancer secretome, where the conditioned media collected from *in vitro* cell cultures (112, 113) is the most common source. Evidently, it is debatable whether cell cultures can replicate the complexity of the tumor microenvironment *in vivo* (114). This notwithstanding, such *in vitro* secretome studies have the advantage of being able to simulate disease models and perturbations in the secretome due to altered physiological parameters or autocrine and/or paracrine secretion (115). Under these conditions, to distinguish between those proteins that are secreted and those that are released into the conditioned media by cell death and proteolysis due to serum-free media culturing conditions, may represent a challenge. Since the concentration of secreted proteins is low, lysis of a low fraction of cells will contaminate the pool of truly secreted proteins due to a high intracellular protein content and thus overshadow the small amount of secreted proteins in the sample (115).

Evidently, *in vivo* and/or *ex vivo* secretome studies are more complex since the microenvironment of the entire tissue is reflected, and due to challenges related to TIF isolation in these situations, there are fewer studies (112, 115). Analysis of fluid harvested from tumor tissue is a powerful approach to bridge the gap between cancer secretomes and tumor biology. Below we address studies performed on tissue fluid.

When studying the *in vivo/ex vivo* secretome, it may be of importance to be able to validate that the proteins in question truly originate from the extracellular fluid phase and thus to differentiate between proteins that are of intracellular origin as recently discussed in more detail (8). Extracellular markers can be applied to validate the origin of the isolated fluid, and specific proteins may also represent intracellular fluid admixture and more specifically, defined intracellular compartments (116).

### (More or less) specific proteins and peptides in TIF

Proteomic profiling of TIF has been performed on samples collected by microdialysis (59, 117), capillary ultrafiltration (68, 69, 118), incubation of tissue in a physiological buffer (65, 116, 119–122), tissue explants/elution (123), and tissue centrifugation (64, 124). Table 2 summarizes TIF studies where human cancer samples have been used as substrate for TIF isolation, and the resulting candidate molecules and validation techniques.

**Table 2:** **Summary of proteomic studies utilizing human tumor interstitial fluid, including candidate proteins that were chosen for validation**.

Analyzed sample	Isolation technique	Samples	Candidates	Validation	Published protein findings	Reference
Mouse colorectal carcinoma (human serum)	Elution	TIF; NIF	MCM4, S100A9	IHC	2172 proteins identified (1958 with human homologs), 52 suggested candidates	(134)
		Serum (control; adenoma; CRC)	CHI3L1, CEA	ELISA		

Hepatocellular carcinoma	Elution	TIF/NIF			381 (TIF) and 245 (NIF) identified proteins, 111 unique for TIF	(116)
		NIF			325 proteins identified in healthy liver	

Hepatocellular carcinoma	Elution	TIF/NIF	sERBB3	Western blot	72 proteins identified	(135)
		Serum (HCC, cirrhosis, chronic hepatitis)	sERBB3, AFP	ELISA		

Renal cell carcinoma	Elution	TIF	ENO2, NNMT	Western blot, SRM	539 proteins identified, 138 up-regulated	(136)
		Serum (patient; normal pool)	ENO2, TSP1	ELISA, SRM		

Ovarian carcinoma	Centrifugation	TIF			769 proteins identified	(64)
		Plasma (patient; control)			124 and 102 proteins identified in patient and control plasma	

Ovarian carcinoma	Elution	TIF/ascites	PRDX1	Western blot	569 proteins identified	(120)
		Serum (EOC; normal/benign)	PRDX1	ELISA		

Ovarian carcinoma	Centrifugation	TIF	WDR1	MRM, SRM WB	6 proteins selected for validation	(124)

Ovarian carcinoma	Elution	TIF/NIF	S100-A8	IHC	58 proteins identified, 1 up-regulated, 5 down-regulated proteins	(137)

Ovarian carcinoma	Elution	TIF; NIF	STIP1, LAP3, TPI1, UCHL1	Western blot, IHC	8 proteins identified	(138)
			BNDF, transferrin	ELISA		
		Serum (patient; control)	STIP1	ELISA		

Breast carcinoma	Elution with biotin	TIF/NIF			93 up-regulated proteins	(139)
		TIF; NIF	CD276	IHC		

Breast carcinoma	Elution	NIF/TIF	Calreticulin, calumenin, TCPT, S100A9	IHC	832 proteins detected, 84 up-regulated proteins	(119)
		TIF; NIF	Calreticulin, CRABP2, CLIC1, EF-1-beta, galectin-1, PRDX2, PD-ECGF, PDI, UCTH5	Tissue microarray	26 of protein candidates present in all patients (gels compared)	

Breast carcinoma	Elution	TIF/NIF	YWHAZ, GDI-1, HNRNPD	Western blot	1324 non-redundant proteins	(121)

Non-small cell lung cancer	Elution	TIF/NIF	PRDX1	Western blot/ELISA	24 proteins differentially expressed	(122)

Colorectal tumor	Explant eluate	TIF/NIF	Desmocollin, fibrinogen γ-chain		32 proteins differentially expressed	(123)

Head and neck squamous cell carcinoma	Capillary ultrafiltration	TIF			525 proteins identified	(118)

Oral squamous cell carcinoma	Microdialysis	TIF; NIF	MMP-8, MMP-9, neurotrypsin, trypsin-1	IHC	217 proteins identified	(117)

*TIF, tumor interstitial fluid; NIF, normal tissue interstitial fluid; MCM4, minichromosome maintenance complex component 4; IHC, immunohistochemistry; CRC, colorectal carcinoma; CHI3L1, chitinase 3-like 1; CEA, carcinoembryonic antigen; ELISA, enzyme-linked immunosorbent assay; sERBB3, secreted receptor tyrosine-kinase erbB-3; HCC, hepatocellular carcinoma; AFP, alpha-fetoprotein; ENO2, enolase 2; NNMT, nicotinamide n-methyltransferase; SRM, selected reaction monitoring; TSP1, thrombospondin-1; PRDX1, peroxiredoxin 1; EOC, epithelial ovarian carcinoma; STIP1, stress-induced phosphoprotein 1; LAP3, leucine aminopeptidase 3; TPI1, triosephosphate isomerase 1; UCHL1, ubiquity carboxyl-terminal esterase L1; BNDF, brain-derived neurotrophic factor; TCPT, translationally controlled tumor protein; CRABP2, cellular retinoic acid-binding protein 2; CLIC1, chloride intracellular channel protein 1; EF-1-beta, elongation factor 1-beta; PRDX2, peroxiredoxin 2; PD-ECGF, platelet-derived endothelial cell growth factor; PDI, protein disulfideisomerase; UCTH5, ubiquitin carboxyl-terminal hydrolase 5; MMP, matrix metalloprotease; WDR1, WD-repeat containing protein 1; YWHAZ, 14-3-3 protein zeta/delta; GDI1, dissociation inhibitor alpha; HNRNPD, AU-rich element RNA-binding protein 1*.

Unfortunately, there are few common validated candidate proteins in the presented TIF studies. Of these, peroxiredoxin 1 and S100A8/9 have been suggested in more than one study, and peroxiredoxin 1 is the most prevalent. It is, however, difficult to integrate results from different studies. The methods for fluid isolation and data collection, analysis, and reporting and the selection of “secreted” proteins may influence the results in the various studies and lead to discrepancies. Even in cell culture studies, where the complexity of an intact tissue is avoided that should reduce biological variation, the trend is similar. In addition, differentially expressed proteins from different cancers and even within one cancer type although in a different model, appear to demonstrate very little overlap (113).

### Common proteins in TIF

In a recent publication (8), we investigated whether common “protein denominators” could be found in TIF, and examined six recently published TIF proteomes in more detail (64, 116–120), all deriving from different cancers and using different TIF extraction methods. Altogether, we found 1805 unique proteins, with 123 proteins (6.8%) discovered in five or six proteomes, and with unique proteins in each proteome; 15% (116), 17% (120), 23% (64), 30% (119), 31% (118), and 59% (117). The 123 common proteins were intracellular enzymes, abundant plasma proteins, and several common cytoplasmic proteins highly conserved in exosomes (125, 126) and several proteins from the 14-3-3 family and peroxiredoxins.

What might appear confusing considering that we were studying TIF, and thus extracellular fluid, was the finding of a substantial fraction of proteins that are classified as intracellular. That is a result of the gene ontology (GO) system, assigning proteins to all the compartments where they have been found. As a consequence, the extracellular compartment plasma will also contain a substantial fraction of proteins classified as intracellular (127). Because TIF contains many of the proteins referred as the exosome “core” proteins (125), intracellular proteins in TIF may actually derive from exosomes. Due to their size, these particles will be sieved off when using techniques involving membranes like microdialysis and ultrafiltration that may again lead to divergent proteomes depending on the isolation techniques, a conclusion that was actually supported by our analysis (8). Based on this evaluation we concluded that there are many common proteins that appear in several proteomes, and moreover that there are many potential unique candidates for each tumor type (8). Another implication of this analysis is that since the isolation method will influence the overall composition of the identified proteome, proteomes from different studies should be evaluated with this in mind. These data might actually suggest that more than one method should be used to isolate TIF in the initial screening for biomarker candidates.

## Summary and Conclusion

In spite of extensive efforts, economical as well as technical, “the inconvenient truth” is that up till now, no biomarker developed by proteomics has been proven to be of benefit for cancer patients (106). The many problems regarding proteomic analysis of serum are well known. This calls for alternative approaches and for new substrates in this endeavor. TIF represents a proximal fluid that may be enriched in tumor specific proteins. It may serve as a new substrate that could be used in a more targeted analysis of the proximal fluids in general. In the present review, we have briefly summarized recent knowledge on the tumor interstitium and the formation and composition of TIF. We have moreover, in particular, addressed proteins secreted to the tumor fluid phase. While several proteomic secretome studies have been performed in cell cultures, only a few studies addressing the TIF proteome have emerged in the recent years, and have been summarized in this article. The isolation of TIF can be challenging *per se*, and the choice of method may have a direct impact on the proteomic results. Unfortunately, even when comparing a fluid that is more proximal to the tumor, i.e., TIF, there are few common validated candidate proteins in the presented TIF studies. There appear to be an unexploited potential in using TIF proteomic data in a functional context. It might appear as a more integrated systems biology biomarker discovery platform should be used. Such a platform should also involve, e.g., cancer cell lines, animal models, tumor tissues, and transcriptomics in addition to proximal fluids (6). Such a strategy will provide new knowledge on tumor biology and hopefully produce new biomarkers or treatment strategies for cancer.

## Conflict of Interest Statement

The authors declare that the research was conducted in the absence of any commercial or financial relationships that could be construed as a potential conflict of interest.

## References

[1] Aukland K Reed RK. Interstitial-lymphatic mechanisms in the control of extracellular fluid volume. Physiol Rev (1993) 73:1–78. 8419962 10.1152/physrev.1993.73.1.1

[2] Randolph GJ Angeli V Swartz MA. Dendritic-cell trafficking to lymph nodes through lymphatic vessels. Nat Rev Immunol (2005) 5:617–28. 10.1038/nri1670 16056255

[3] Baronzio G Schwartz L Kiselevsky M Guais A Sanders E Milanesi G Tumor interstitial fluid as modulator of cancer inflammation, thrombosis, immunity and angiogenesis. Anticancer Res (2012) 32:405–14. 22287726

[4] Wiig H Tenstad O Iversen PO Kalluri R Bjerkvig R. Interstitial fluid: the overlooked component of the tumor microenvironment? Fibrogenesis Tissue Repair (2010) 3:12. 10.1186/1755-1536-3-12 20653943 PMC2920231

[5] Baronzio G Parmar G Baronzio M Kiselevsky M. Tumor interstitial fluid: proteomic determination as a possible source of biomarkers. Cancer Genomics Proteomics (2014) 11:225–37. 25331795

[6] Kulasingam V Pavlou MP Diamandis EP. Integrating high-throughput technologies in the quest for effective biomarkers for ovarian cancer. Nat Rev Cancer (2010) 10:371–8. 10.1038/nrc2831 20383179

[7] Wiig H Swartz MA. Interstitial fluid and lymph formation and transport: physiological regulation and roles in inflammation and cancer. Physiol Rev (2012) 92:1005–60. 10.1152/physrev.00037.2011 22811424

[8] Haslene-Hox H Tenstad O Wiig H. Interstitial fluid-a reflection of the tumor cell microenvironment and secretome. Biochim Biophys Acta (2013) 1834:2336–46. 10.1016/j.bbapap.2013.01.028 23376185

[9] Kalluri R Zeisberg M Fibroblasts in cancer. Nat Rev Cancer (2006) 6:392–401. 10.1038/nrc1877 16572188

[10] Mueller MM Fusenig NE. Friends or foes – bipolar effects of the tumour stroma in cancer. Nat Rev Cancer (2004) 4:839–49. 10.1038/nrc1477 15516957

[11] Liotta LA Kohn EC The microenvironment of the tumour-host interface. Nature (2001) 411:375–9. 10.1038/35077241 11357145

[12] Sund M Kalluri R. Tumor stroma derived biomarkers in cancer. Cancer Metastasis Rev (2009) 28:177–83. 10.1007/s10555-008-9175-2 19259624 PMC4476244

[13] Pickup MW Mouw JK Weaver VM. The extracellular matrix modulates the hallmarks of cancer. EMBO Rep (2014) 15:1243–53. 10.15252/embr.201439246 25381661 PMC4264927

[14] Miles FL Sikes RA. Insidious changes in stromal matrix fuel cancer progression. Mol Cancer Res (2014) 12:297–312. 10.1158/1541-7786.MCR-13-0535 24452359 PMC4066664

[15] Lu P Weaver VM Werb Z The extracellular matrix: a dynamic niche in cancer progression. J Cell Biol (2012) 196:395–406. 10.1083/jcb.201102147 22351925 PMC3283993

[16] Junttila MR de Sauvage FJ. Influence of tumour micro-environment heterogeneity on therapeutic response. Nature (2013) 501:346–54. 10.1038/nature12626 24048067

[17] Sica A Mantovani A. Macrophage plasticity and polarization: in vivo veritas. J Clin Invest (2012) 122:787–95. 10.1172/JCI59643 22378047 PMC3287223

[18] Woo SR Corrales L Gajewski TF Innate immune recognition of cancer. Annu Rev Immunol (2015) 33:445–74. 10.1146/annurev-immunol-032414-112043 25622193

[19] Wynn TA Chawla A Pollard JW Macrophage biology in development, homeostasis and disease. Nature (2013) 496:445–55. 10.1038/nature12034 23619691 PMC3725458

[20] Martinez FO Gordon S. The M1 and M2 paradigm of macrophage activation: time for reassessment. F1000Prime Rep (2014) 6:13. 10.12703/P6-13 24669294 PMC3944738

[21] Fridman WH Pages F Sautes-Fridman C Galon J. The immune contexture in human tumours: impact on clinical outcome. Nat Rev Cancer (2012) 12:298–306. 10.1038/nrc3245 22419253

[22] Gajewski TF Schreiber H Fu YX. Innate and adaptive immune cells in the tumor microenvironment. Nat Immunol (2013) 14:1014–22. 10.1038/ni.2703 24048123 PMC4118725

[23] Sinha P Clements VK Bunt SK Albelda SM Ostrand-Rosenberg S. Cross-talk between myeloid-derived suppressor cells and macrophages subverts tumor immunity toward a type 2 response. J Immunol (2007) 179:977–83. 10.4049/jimmunol.179.2.977 17617589

[24] Mahfouz SM Chevallier M Grimaud JA Distribution of the major connective matrix components of the stromal reaction in breast carcinoma. An immunohistochemical study. Cell Mol Biol (1987) 33:453–67. 3311367

[25] Takeuchi J Sobue M Sato E Shamoto M Miura K. Variation in glycosaminoglycan components of breast tumors. Cancer Res (1976) 36:2133–9. 179697

[26] Yeo TK Brown L Dvorak HF. Alterations in proteoglycan synthesis common to healing wounds and tumors. Am J Pathol (1991) 138:1437–50. 1711290 PMC1886391

[27] Ronnov-Jessen L Petersen OW Bissell MJ. Cellular changes involved in conversion of normal to malignant breast: importance of the stromal reaction. Physiol Rev (1996) 76:69–125. 8592733 10.1152/physrev.1996.76.1.69

[28] Brown LF Guidi AJ Schnitt SJ Van De Water L Iruela-Arispe ML Yeo TK Vascular stroma formation in carcinoma in situ, invasive carcinoma, and metastatic carcinoma of the breast. Clin Cancer Res (1999) 5:1041–56. 10353737

[29] Fukumura D Xavier R Sugiura T Chen Y Park EC Lu N Tumor induction of VEGF promoter activity in stromal cells. Cell (1998) 94:715–25. 10.1016/S0092-8674(00)81731-6 9753319

[30] Dvorak HF Sioussat TM Brown LF Berse B Nagy JA Sotrel A Distribution of vascular permeability factor (vascular endothelial growth factor) in tumors: concentration in tumor blood vessels. J Exp Med (1991) 174:1275–8. 10.1084/jem.174.5.1275 1940805 PMC2118980

[31] Senger DR Galli SJ Dvorak AM Perruzzi CA Harvey VS Dvorak HF Tumor cells secrete a vascular permeability factor that promotes accumulation of ascites fluid. Science (1983) 219:983–5. 10.1126/science.6823562 6823562

[32] Dvorak HF Tumors: wounds that do not heal. Similarities between tumor stroma generation and wound healing. N Engl J Med (1986) 315:1650–9. 10.1056/NEJM198612253152606 3537791

[33] Mantovani A Allavena P Sica A Balkwill F. Cancer-related inflammation. Nature (2008) 454:436–44. 10.1038/nature07205 18650914

[34] Christiansen A Detmar M Lymphangiogenesis and cancer. Genes Cancer (2011) 2:1146–58. 10.1177/1947601911423028 22866206 PMC3411123

[35] Mouw JK Ou G Weaver VM. Extracellular matrix assembly: a multiscale deconstruction. Nat Rev Mol Cell Biol (2014) 15:771–85. 10.1038/nrm3902 25370693 PMC4682873

[36] Bonnans C Chou J Werb Z. Remodelling the extracellular matrix in development and disease. Nat Rev Mol Cell Biol (2014) 15:786–801. 10.1038/nrm3904 25415508 PMC4316204

[37] Starling EH On the absorption of fluids from the connective tissue spaces. J Physiol (1896) 19:312–26. 10.1113/jphysiol.1896.sp000596 PMC1512609 16992325

[38] Levick JR Michel CC. Microvascular fluid exchange and the revised starling principle. Cardiovasc Res (2010) 87:198–210. 10.1093/cvr/cvq062 20200043

[39] Fukumura D Jain RK. Tumor microenvironment abnormalities: causes, consequences, and strategies to normalize. J Cell Biochem (2007) 101:937–49. 10.1002/jcb.21187 17171643

[40] Heldin CH Rubin K Pietras K Ostman A. High interstitial fluid pressure – an obstacle in cancer therapy. Nat Rev Cancer (2004) 4:806–13. 10.1038/nrc1456 15510161

[41] Goel S Duda DG Xu L Munn LL Boucher Y Fukumura D Normalization of the vasculature for treatment of cancer and other diseases. Physiol Rev (2011) 91:1071–121. 10.1152/physrev.00038.2010 21742796 PMC3258432

[42] Wiig H. Evaluation of methodologies for measurement of interstitial fluid pressure (Pi): physiological implications of recent Pi data. Crit Rev Biomed Eng (1990) 18:27–54. 2204514

[43] Provenzano PP Cuevas C Chang AE Goel VK Von Hoff DD Hingorani SR. Enzymatic targeting of the stroma ablates physical barriers to treatment of pancreatic ductal adenocarcinoma. Cancer Cell (2012) 21:418–29. 10.1016/j.ccr.2012.01.007 22439937 PMC3371414

[44] Khawar IA Kim JH Kuh HJ. Improving drug delivery to solid tumors: priming the tumor microenvironment. J Control Release (2015) 201:78–89. 10.1016/j.jconrel.2014.12.018 25526702

[45] Ariffin AB Forde PF Jahangeer S Soden DM Hinchion J. Releasing pressure in tumors: what do we know so far and where do we go from here? A review. Cancer Res (2014) 74:2655–62. 10.1158/0008-5472.CAN-13-3696 24778418

[46] Azzi S Hebda JK Gavard J. Vascular permeability and drug delivery in cancers. Front Oncol (2013) 3:211. 10.3389/fonc.2013.00211 23967403 PMC3744053

[47] Dvorak HF Brown LF Detmar M Dvorak AM. Vascular permeability factor/vascular endothelial growth factor, microvascular hyperpermeability, and angiogenesis. Am J Pathol (1995) 146:1029–39. 7538264 PMC1869291

[48] Baluk P Morikawa S Haskell A Mancuso M McDonald DM. Abnormalities of basement membrane on blood vessels and endothelial sprouts in tumors. Am J Pathol (2003) 163:1801–15. 10.1016/S0002-9440(10)63540-7 14578181 PMC1892429

[49] Wiig H Tveit E Hultborn R Reed RK Weiss L. Interstitial fluid pressure in DMBA-induced rat mammary tumours. Scand J Clin Lab Invest (1982) 42:159–64. 10.3109/00365518209168068 6813944

[50] Stohrer M Boucher Y Stangassinger M Jain RK. Oncotic pressure in solid tumors is elevated. Cancer Res (2000) 60:4251–5. 10945638

[51] Wiig H Aukland K Tenstad O. Isolation of interstitial fluid from rat mammary tumors by a centrifugation method. Am J Physiol Heart Circ Physiol (2003) 284:H416–24. 10.1152/ajpheart.00327.2002 12388326

[52] Leu AJ Berk DA Lymboussaki A Alitalo K Jain RK. Absence of functional lymphatics within a murine sarcoma: a molecular and functional evaluation. Cancer Res (2000) 60:4324–7. 10969769

[53] Padera TP Kadambi A di Tomaso E Carreira CM Brown EB Boucher Y Lymphatic metastasis in the absence of functional intratumor lymphatics. Science (2002) 296:1883–6. 10.1126/science.1071420 11976409

[54] Padera TP Stoll BR Tooredman JB Capen D di Tomaso E Jain RK. Pathology: cancer cells compress intratumour vessels. Nature (2004) 427:695. 10.1038/427695a 14973470

[55] Klemm F Joyce JA Microenvironmental regulation of therapeutic response in cancer. Trends Cell Biol (2015) 25:198–213. 10.1016/j.tcb.2014.11.006 25540894 PMC5424264

[56] Alitalo K Tammela T Petrova TV. Lymphangiogenesis in development and human disease. Nature (2005) 438:946–53. 10.1038/nature04480 16355212

[57] Thiele W Sleeman JP. Tumor-induced lymphangiogenesis: a target for cancer therapy? J Biotechnol (2006) 124:224–41. 10.1016/j.jbiotec.2006.01.007 16497404

[58] Stacker SA Caesar C Baldwin ME Thornton GE Williams RA Prevo R VEGF-D promotes the metastatic spread of tumor cells via the lymphatics. Nat Med (2001) 7:186–91. 10.1038/84635 11175849

[59] Xu BJ Yan W Jovanovic B Shaw AK An QA Eng J Microdialysis combined with proteomics for protein identification in breast tumor microenvironment in vivo. Cancer Microenviron (2010) 4:61–71. 10.1007/s12307-010-0046-3 21505562 PMC3047629

[60] Wiig H. Cornea fluid dynamics. I: measurement of hydrostatic and colloid osmotic pressure in rabbits. Exp Eye Res (1989) 49:1015–30. 10.1016/S0014-4835(89)80023-5 2612582

[61] Aukland K. Distribution volumes and macromolecular mobility in rat tail tendon interstitium. Am J Physiol (1991) 260:H409–19. 1996684 10.1152/ajpheart.1991.260.2.H409

[62] Choi J Credit K Henderson K Deverkadra R He Z Wiig H Intraperitoneal immunotherapy for metastatic ovarian carcinoma: resistance of intratumoral collagen to antibody penetration. Clin Cancer Res (2006) 12:1906–12. 10.1158/1078-0432.CCR-05-2141 16551876

[63] Salnikov AV Heldin NE Stuhr LB Wiig H Gerber H Reed RK Inhibition of carcinoma cell-derived VEGF reduces inflammatory characteristics in xenograft carcinoma. Int J Cancer (2006) 119:2795–802. 10.1002/ijc.22217 17019708

[64] Haslene-Hox H Oveland E Berg KC Kolmannskog O Woie K Salvesen HB A new method for isolation of interstitial fluid from human solid tumors applied to proteomic analysis of ovarian carcinoma tissue. PLoS One (2011) 6:e19217. 10.1371/journal.pone.0019217 21541282 PMC3082557

[65] Celis JE Gromov P Cabezon T Moreira JM Ambartsumian N Sandelin K Proteomic characterization of the interstitial fluid perfusing the breast tumor microenvironment: a novel resource for biomarker and therapeutic target discovery. Mol Cell Proteomics (2004) 3:327–44. 10.1074/mcp.M400009-MCP200 14754989

[66] Leegsma-Vogt G Janle E Ash SR Venema K Korf J. Utilization of in vivo ultrafiltration in biomedical research and clinical applications. Life Sci (2003) 73:2005–18. 10.1016/S0024-3205(03)00569-1 12899925

[67] Schneiderheinze JM Hogan BL. Selective in vivo and in vitro sampling of proteins using miniature ultrafiltration sampling probes. Anal Chem (1996) 68:3758–62. 10.1021/ac960309u 21619247

[68] Huang CM Ananthaswamy HN Barnes S Ma Y Kawai M Elmets CA. Mass spectrometric proteomics profiles of in vivo tumor secretomes: capillary ultrafiltration sampling of regressive tumor masses. Proteomics (2006) 6:6107–16. 10.1002/pmic.200600287 17051643

[69] Yang S Huang CM Recent advances in protein profiling of tissues and tissue fluids. Expert Rev Proteomics (2007) 4:515–29. 10.1586/14789450.4.4.515 17705709

[70] Dabrosin C. Microdialysis – an in vivo technique for studies of growth factors in breast cancer. Front Biosci (2005) 10:1329–35. 10.2741/1622 15769628

[71] Benjamin RK Hochberg FH Fox E Bungay PM Elmquist WF Stewart CF Review of microdialysis in brain tumors, from concept to application: first annual Carolyn Frye-Halloran symposium. Neuro Oncol (2004) 6:65–74. 10.1215/S1152851703000103 14769143 PMC1871970

[72] Brunner M Muller M. Microdialysis: an in vivo approach for measuring drug delivery in oncology. Eur J Clin Pharmacol (2002) 58:227–34. 10.1007/s00228-002-0475-0 12136367

[73] Liu L Zhang X Lou Y Rao Y Zhang X. Cerebral microdialysis in glioma studies, from theory to application. J Pharm Biomed Anal (2014) 96:77–89. 10.1016/j.jpba.2014.03.026 24747145

[74] Blakeley J Portnow J. Microdialysis for assessing intratumoral drug disposition in brain cancers: a tool for rational drug development. Expert Opin Drug Metab Toxicol (2010) 6:1477–91. 10.1517/17425255.2010.523420 20969450 PMC3994531

[75] Zhou Q Gallo JM. In vivo microdialysis for PK and PD studies of anticancer drugs. AAPS J (2005) 7:E659–67. 10.1208/aapsj070366 16353942 PMC2751268

[76] Ao X Stenken JA Microdialysis sampling of cytokines. Methods (2006) 38:331–41. 10.1016/j.ymeth.2005.11.012 16487724

[77] Clough GF. Microdialysis of large molecules. AAPS J (2005) 7:E686–92. 10.1208/aapsj070369 16353945 PMC2751271

[78] Hersini KJ Melgaard L Gazerani P Petersen LJ. Microdialysis of inflammatory mediators in the skin: a review. Acta Derm Venereol (2014) 94:501–11. 10.2340/00015555-1878 24890140

[79] Bendrik C Dabrosin C. Estradiol increases IL-8 secretion of normal human breast tissue and breast cancer in vivo. J Immunol (2009) 182:371–8. 10.4049/jimmunol.182.1.371 19109168

[80] Wibom C Surowiec I Moren L Bergstrom P Johansson M Antti H Metabolomic patterns in glioblastoma and changes during radiotherapy: a clinical microdialysis study. J Proteome Res (2010) 9:2909–19. 10.1021/pr901088r 20302353

[81] Gullino PM Clark SH Grantham FH The interstitial fluid of solid tumors. Cancer Res (1964) 24:780–98. 14190544

[82] Jain RK Shah SA Finney PL. Continuous noninvasive monitoring of pH and temperature in rat walker 256 carcinoma during normoglycemia and hyperglycemia. J Natl Cancer Inst (1984) 73:429–36. 6589435 10.1093/jnci/73.2.429

[83] Grahovac J Wells A. Matrikine and matricellular regulators of EGF receptor signaling on cancer cell migration and invasion. Lab Invest (2014) 94:31–40. 10.1038/labinvest.2013.132 24247562 PMC4038324

[84] Ricard-Blum S Salza R. Matricryptins and matrikines: biologically active fragments of the extracellular matrix. Exp Dermatol (2014) 23:457–63. 10.1111/exd.12435 24815015

[85] Monboisse JC Oudart JB Ramont L Brassart-Pasco S Maquart FX. Matrikines from basement membrane collagens: a new anti-cancer strategy. Biochim Biophys Acta (2014) 1840:2589–98. 10.1016/j.bbagen.2013.12.029 24406397

[86] Voloshin T Fremder E Shaked Y. Small but mighty: microparticles as mediators of tumor progression. Cancer Microenviron (2014) 7:11–21. 10.1007/s12307-014-0144-8 24705797 PMC4150874

[87] Andaloussi SE Mager I Breakefield XO Wood MJ. Extracellular vesicles: biology and emerging therapeutic opportunities. Nat Rev Drug Discov (2013) 12:347–57. 10.1038/nrd3978 23584393

[88] Colombo M Raposo G Thery C. Biogenesis, secretion, and intercellular interactions of exosomes and other extracellular vesicles. Annu Rev Cell Dev Biol (2014) 30:255–89. 10.1146/annurev-cellbio-101512-122326 25288114

[89] Antonyak MA Cerione RA. Microvesicles as mediators of intercellular communication in cancer. Methods Mol Biol (2014) 1165:147–73. 10.1007/978-1-4939-0856-1_11 24839024

[90] Mause SF Weber C. Microparticles: protagonists of a novel communication network for intercellular information exchange. Circ Res (2010) 107:1047–57. 10.1161/CIRCRESAHA.110.226456 21030722

[91] Morel O Morel N Jesel L Freyssinet JM Toti F. Microparticles: a critical component in the nexus between inflammation, immunity, and thrombosis. Semin Immunopathol (2011) 33:469–86. 10.1007/s00281-010-0239-3 21866419

[92] D’Souza-Schorey C Clancy JW. Tumor-derived microvesicles: shedding light on novel microenvironment modulators and prospective cancer biomarkers. Genes Dev (2012) 26:1287–99. 10.1101/gad.192351.112 22713869 PMC3387656

[93] D’Asti E Garnier D Lee TH Montermini L Meehan B Rak J. Oncogenic extracellular vesicles in brain tumor progression. Front Physiol (2012) 3:294. 10.3389/fphys.2012.00294 22934045 PMC3429065

[94] Loyer X Vion AC Tedgui A Boulanger CM. Microvesicles as cell-cell messengers in cardiovascular diseases. Circ Res (2014) 114:345–53. 10.1161/CIRCRESAHA.113.300858 24436430

[95] Julich H Willms A Lukacs-Kornek V Kornek M. Extracellular vesicle profiling and their use as potential disease specific biomarker. Front Immunol (2014) 5:413. 10.3389/fimmu.2014.00413 25225495 PMC4150251

[96] Shao H Chung J Balaj L Charest A Bigner DD Carter BS Protein typing of circulating microvesicles allows real-time monitoring of glioblastoma therapy. Nat Med (2012) 18:1835–40. 10.1038/nm.2994 23142818 PMC3518564

[97] Iversen PO Wiig H. Tumor necrosis factor alpha and adiponectin in bone marrow interstitial fluid from patients with acute myeloid leukemia inhibit normal hematopoiesis. Clin Cancer Res (2005) 11:6793–9. 10.1158/1078-0432.CCR-05-1033 16203766

[98] Haslene-Hox H Madani A Berg KCG Woie K Salvesen HB Wiig H Quantification of the concentration gradient of biomarkers between ovarian carcinoma interstitial fluid and blood. Biochim Biophys Acta Clin (2014) 2:18–23. 10.1016/j.bbacli.2014.08.002 PMC4633919 26673827

[99] Swartz MA Lund AW. Lymphatic and interstitial flow in the tumour microenvironment: linking mechanobiology with immunity. Nat Rev Cancer (2012) 12:210–9. 10.1038/nrc3186 22362216

[100] Shields JD Fleury ME Yong C Tomei AA Randolph GJ Swartz MA. Autologous chemotaxis as a mechanism of tumor cell homing to lymphatics via interstitial flow and autocrine CCR7 signaling. Cancer Cell (2007) 11:526–38. 10.1016/j.ccr.2007.04.020 17560334

[101] Sagstad SJ Oveland E Karlsen TV Haslene-Hox H Tenstad O Wiig H. Age-related changes in rat dermal extracellular matrix composition affect the distribution of plasma proteins as a function of size and charge. Am J Physiol Heart Circ Physiol (2015) 308:H29–38. 10.1152/ajpheart.00545.2014 25362136

[102] Haslene-Hox H Oveland E Woie K Salvesen HB Tenstad O Wiig H. Distribution volumes of macromolecules in human ovarian and endometrial cancers-effects of extracellular matrix structure. Am J Physiol Heart Circ Physiol (2015) 308:H18–28. 10.1152/ajpheart.00672.2014 25380817

[103] Polanski M Anderson NL. A list of candidate cancer biomarkers for targeted proteomics. Biomark Insights (2007) 1:1–48. 19690635 PMC2716785

[104] Rifai N Gillette MA Carr SA. Protein biomarker discovery and validation: the long and uncertain path to clinical utility. Nat Biotechnol (2006) 24:971–83. 10.1038/nbt1235 16900146

[105] Veenstra TD. Global and targeted quantitative proteomics for biomarker discovery. J Chromatogr B Analyt Technol Biomed Life Sci (2007) 847:3–11. 10.1016/j.jchromb.2006.09.004 17023222

[106] Kondo T. Inconvenient truth: cancer biomarker development by using proteomics. Biochim Biophys Acta (2014) 1844:861–5. 10.1016/j.bbapap.2013.07.009 23896458

[107] Gromov P Gromova I Olsen CJ Timmermans-Wielenga V Talman ML Serizawa RR Tumor interstitial fluid – a treasure trove of cancer biomarkers. Biochim Biophys Acta (2013) 1834:2259–70. 10.1016/j.bbapap.2013.01.013 23416532

[108] Sedlaczek P Frydecka I Gabrys M Van Dalen A Einarsson R Harlozinska A. Comparative analysis of CA125, tissue polypeptide specific antigen, and soluble interleukin-2 receptor alpha levels in sera, cyst, and ascitic fluids from patients with ovarian carcinoma. Cancer (2002) 95:1886–93. 10.1002/cncr.10917 12404282

[109] Simpson RJ Bernhard OK Greening DW Moritz RL. Proteomics-driven cancer biomarker discovery: looking to the future. Curr Opin Chem Biol (2008) 12:72–7. 10.1016/j.cbpa.2008.02.010 18295612

[110] Teng PN Bateman NW Hood BL Conrads TP. Advances in proximal fluid proteomics for disease biomarker discovery. J Proteome Res (2010) 9:6091–100. 10.1021/pr100904q 21028795

[111] Gronborg M Kristiansen TZ Iwahori A Chang R Reddy R Sato N Biomarker discovery from pancreatic cancer secretome using a differential proteomic approach. Mol Cell Proteomics (2006) 5:157–71. 10.1074/mcp.M500178-MCP200 16215274

[112] Xue H Lu B Lai M. The cancer secretome: a reservoir of biomarkers. J Transl Med (2008) 6:52. 10.1186/1479-5876-6-52 18796163 PMC2562990

[113] Makridakis M Vlahou A. Secretome proteomics for discovery of cancer biomarkers. J Proteomics (2010) 73:2291–305. 10.1016/j.jprot.2010.07.001 20637910

[114] Karagiannis GS Pavlou MP Diamandis EP. Cancer secretomics reveal pathophysiological pathways in cancer molecular oncology. Mol Oncol (2010) 4:496–510. 10.1016/j.molonc.2010.09.001 20934395 PMC5527923

[115] Stastna M Van Eyk JE. Secreted proteins as a fundamental source for biomarker discovery. Proteomics (2012) 12:722–35. 10.1002/pmic.201100346 22247067 PMC3517109

[116] Sun W Ma J Wu S Yang D Yan Y Liu K Characterization of the liver tissue interstitial fluid (TIF) proteome indicates potential for application in liver disease biomarker discovery. J Proteome Res (2010) 9:1020–31. 10.1021/pr9009172 20038183

[117] Hardt M Lam DK Dolan JC Schmidt BL. Surveying proteolytic processes in human cancer microenvironments by microdialysis and activity-based mass spectrometry. Proteomics Clin Appl (2011) 5:636–43. 10.1002/prca.201100015 22262628 PMC3470480

[118] Stone MD Odland RM McGowan T Onsongo G Tang C Rhodus NL Novel in situ collection of tumor interstitial fluid from a head and neck squamous carcinoma reveals a unique proteome with diagnostic potential. Clin Proteomics (2010) 6:75–82. 10.1007/s12014-010-9050-3 20930922 PMC2937136

[119] Gromov P Gromova I Bunkenborg J Cabezon T Moreira JM Timmermans-Wielenga V Up-regulated proteins in the fluid bathing the tumour cell microenvironment as potential serological markers for early detection of cancer of the breast. Mol Oncol (2010) 4:65–89. 10.1016/j.molonc.2009.11.003 20005186 PMC5527961

[120] Hoskins ER Hood BL Sun M Krivak TC Edwards RP Conrads TP. Proteomic analysis of ovarian cancer proximal fluids: validation of elevated peroxiredoxin 1 in patient peripheral circulation. PLoS One (2011) 6:e25056. 10.1371/journal.pone.0025056 21980378 PMC3184097

[121] Raso C Cosentino C Gaspari M Malara N Han X McClatchy D Characterization of breast cancer interstitial fluids by TmT labeling, LTQ-orbitrap velos mass spectrometry, and pathway analysis. J Proteome Res (2012) 11:3199–210. 10.1021/pr2012347 22563702 PMC3562392

[122] Li S Wang R Zhang M Wang L Cheng S. Proteomic analysis of non-small cell lung cancer tissue interstitial fluids. World J Surg Oncol (2013) 11:173. 10.1186/1477-7819-11-173 23914992 PMC3751644

[123] Shi HJ Stubbs R Hood K. Characterization of de novo synthesized proteins released from human colorectal tumour explants. Electrophoresis (2009) 30:2442–53. 10.1002/elps.200800767 19639566

[124] Haslene-Hox H Oveland E Woie K Salvesen HB Wiig H Tenstad O. Increased WD-repeat containing protein 1 in interstitial fluid from ovarian carcinomas shown by comparative proteomic analysis of malignant and healthy gynecological tissue. Biochim Biophys Acta (2013) 1834:2347–59. 10.1016/j.bbapap.2013.05.011 23707566

[125] Raimondo F Morosi L Chinello C Magni F Pitto M. Advances in membranous vesicle and exosome proteomics improving biological understanding and biomarker discovery. Proteomics (2011) 11:709–20. 10.1002/pmic.201000422 21241021

[126] Simpson RJ Lim JW Moritz RL Mathivanan S. Exosomes: proteomic insights and diagnostic potential. Expert Rev Proteomics (2009) 6:267–83. 10.1586/epr.09.17 19489699

[127] Pieper R Gatlin CL Makusky AJ Russo PS Schatz CR Miller SS The human serum proteome: display of nearly 3700 chromatographically separated protein spots on two-dimensional electrophoresis gels and identification of 325 distinct proteins. Proteomics (2003) 3:1345–64. 10.1002/pmic.200300449 12872236

[128] Wiig H Keskin D Kalluri R. Interaction between the extracellular matrix and lymphatics: consequences for lymphangiogenesis and lymphatic function. Matrix Biol (2010) 29:645–56. 10.1016/j.matbio.2010.08.001 20727409 PMC3992865

[129] Gullino PM Grantham FH Smith SH Haggerty AC Modifications of the acid-base status of the internal milieu of tumors. J Natl Cancer Inst (1965) 34:857–69. 4284033

[130] Helmlinger G Sckell A Dellian M Forbes NS Jain RK. Acid production in glycolysis-impaired tumors provides new insights into tumor metabolism. Clin Cancer Res (2002) 8:1284–91. 11948144

[131] Helmlinger G Yuan F Dellian M Jain RK. Interstitial pH and pO2 gradients in solid tumors in vivo: high-resolution measurements reveal a lack of correlation. Nat Med (1997) 3:177–82. 10.1038/nm0297-177 9018236

[132] Hockel M Schlenger K Hockel S Vaupel P. Hypoxic cervical cancers with low apoptotic index are highly aggressive. Cancer Res (1999) 59:4525–8. 10493500

[133] Vaupel P Mayer A. Hypoxia in cancer: significance and impact on clinical outcome. Cancer Metastasis Rev (2007) 26:225–39. 10.1007/s10555-007-9055-1 17440684

[134] Fijneman RJ de Wit M Pourghiasian M Piersma SR Pham TV Warmoes MO Proximal fluid proteome profiling of mouse colon tumors reveals biomarkers for early diagnosis of human colorectal cancer. Clin Cancer Res (2012) 18:2613–24. 10.1158/1078-0432.CCR-11-1937 22351690

[135] Hsieh SY He JR Yu MC Lee WC Chen TC Lo SJ Secreted ERBB3 isoforms are serum markers for early hepatoma in patients with chronic hepatitis and cirrhosis. J Proteome Res (2011) 10:4715–24. 10.1021/pr200519q 21877752

[136] Teng PN Hood BL Sun M Dhir R Conrads TP. Differential proteomic analysis of renal cell carcinoma tissue interstitial fluid. J Proteome Res (2011) 10:1333–42. 10.1021/pr101074p 21142074

[137] Cortesi L Rossi E Della Casa L Barchetti A Nicoli A Piana S Protein expression patterns associated with advanced stage ovarian cancer. Electrophoresis (2011) 32:1992–2003. 10.1002/elps.201000654 21728179

[138] Wang TH Chao A Tsai CL Chang CL Chen SH Lee YS Stress-induced phosphoprotein 1 as a secreted biomarker for human ovarian cancer promotes cancer cell proliferation. Mol Cell Proteomics (2010) 9:1873–84. 10.1074/mcp.M110.000802 20501939 PMC2938116

[139] Turtoi A Dumont B Greffe Y Blomme A Mazzucchelli G Delvenne P Novel comprehensive approach for accessible biomarker identification and absolute quantification from precious human tissues. J Proteome Res (2011) 10:3160–82. 10.1021/pr200212r 21534635

